# Neuropeptides and the Nodes of Ranvier in Cranial Headaches

**DOI:** 10.3389/fphys.2021.820037

**Published:** 2022-01-12

**Authors:** Jacob C. A. Edvinsson, Kristian A. Haanes, Lars Edvinsson

**Affiliations:** ^1^Department of Clinical Experimental Research, Rigshospitalet-Glostrup, Copenhagen, Denmark; ^2^Department of Drug Design and Pharmacology, University of Copenhagen, Copenhagen, Denmark; ^3^Department of Internal Medicine, Lund University, Lund, Sweden

**Keywords:** trigeminal ganglion, nodes of Ranvier, Remak bundles, vascular neuroeffector site, nerve entry zone, Redlich-Obersteiner zone

## Abstract

The trigeminovascular system (TGV) comprise of the trigeminal ganglion with neurons and satellite glial cells, with sensory unmyelinated C-fibers and myelinated Aδ-fibers picking up information from different parts of the head and sending signals to the brainstem and the central nervous system. In this review we discuss aspects of signaling at the distal parts of the sensory fibers, the extrasynaptic signaling between C-fibers and Aδ-fibers, and the contact between the trigeminal fibers at the nerve root entry zone where they transit into the CNS. We also address the possible role of the neuropeptides calcitonin gene-related peptide (CGRP), the neurokinin family and pituitary adenylyl cyclase-activating polypeptide 38 (PACAP-38), all found in the TGV system together with their respective receptors. Elucidation of the expression and localization of neuropeptides and their receptors in the TGV system may provide novel ways to understand their roles in migraine pathophysiology and suggest novel ways for treatment of migraine patients.

## Introduction

There is strong evidence for a role of calcitonin gene-related peptide (CGRP) and the calcitonin receptor-like receptor/receptor activity-modifying protein 1 (CLR/RAMP1, the CGRP receptor elements) in migraine pathophysiology (Edvinsson et al., 2018). CGRP is expressed in small to medium sized neurons in the trigeminal ganglion (TG) that project sensory, afferent non-myelinated C-fibers to both intra- and extracranial structures and centrally to the trigeminal nucleus caudalis (TNC; Edvinsson and Uddman, 2005; Eftekhari et al., 2010, 2013). The TG also harbors larger neurons and satellite glial cells (SGCs) which express the CGRP receptor. These neurons project thinly myelinated afferent sensory Aδ-fibers often in close relation to the C-fibers to the same regions as the C-fibers (Eftekhari et al., 2010, 2013). Early work demonstrated that CGRP is released from the trigeminovascular (TGV) system while more recent work has revealed CGRP release in attacks of primary headaches from sites such as the TG with neuronal bodies (soma), the trigeminal fibers (soma-free parts) and the dura mater and cranial vasculature which represent the most distal parts of the sensory nerve fibers (Edvinsson et al., 2018). Since neither CGRP nor CGRP receptor blocking drugs can pass the blood-brain barrier (BBB) freely, the likely site of anti-migraine action of the novel gepants and monoclonal antibodies (mAbs) is within the TGV system which lacks a BBB (Lundblad et al., 2015).

**FIGURE 1:**
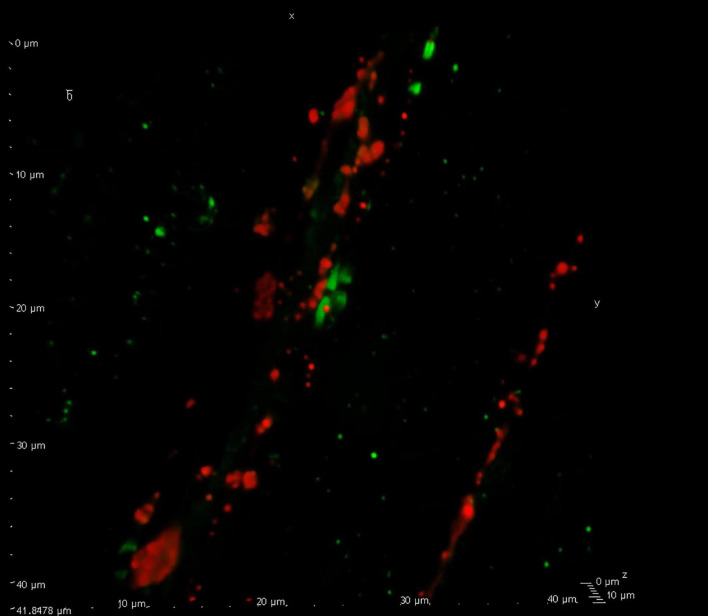
Confocal visualization of the node of Ranvier. Confocal microscopy displaying calcitonin gene-related peptide (CGRP) (red) immunreactive C-fiber boutons flanked by two Aδ-fibers with their paranodal regions marked with contactin associated protein 1 (CASPR, green). This close relationship between the two fiber types display a point of interaction for CGRP signaling within the trigeminovascular (TGV) system (see Supplementary Video). For experimental details on antibodies etc., see Edvinsson et al. (2019).

The aim of this review is to discuss in some detail the close relation between the C- and Aδ-fibers, occurring when the boutons align with or come close to proximal nodes of Ranvier, proposing the hypothesis that this might be a site of action of headache medications. Current data point toward the intimate location here between CGRP containing C-fiber boutons in proximity to the nodes of Ranvier (Figure 1 and Supplementary Video) where recent work has identified location of CGRP receptors on the Aδ-fibers (Edvinsson et al., 2019). Substance P (neurokinin family) (Edvinsson et al., 2021) and the pituitary adenylyl cyclase-activating polypeptide - 38 (PACAP-38) and their receptors in relation to the CGRP system (Edvinsson J. C. A. et al., 2020), has also been investigated in light of the node of Ranvier, and discussed below.

## Expression and Release of Calcitonin Gene-Related Peptide, Substance P, and Pituitary Adenylyl Cyclase-Activating Polypeptide From the Trigeminovascular System

Studies have examined the release of CGRP, substance P, and PACAP from neuron rich regions the TG, neuron soma poor regions of the TG and the dura mater with its vasculature (Edvinsson J. C. A. et al., 2020; Edvinsson et al., 2021). Along the unmyelinated C-fibers from the TG in both central and peripheral direction the thin C-fibers often appear as a pearl necklace with the “pearls” being the so-called “boutons,” sometimes described also as a pattern “en-passant” in the fibers with accumulations of neurotransmitters. Immunohistochemistry revealed that the C-fibers and TG neurons contain a rich supply of CGRP, while about 15% of these contain substance P (Edvinsson et al., 2021) and PACAP (Edvinsson J. C. A. et al., 2020); even fewer contain neurokinins A and B. Our analysis revealed a minor content of substance P in the CGRP containing C-fiber boutons while there was no observable presence of PACAP. Detailed analysis of other peptides of the CGRP family such as amylin and adrenomedullin revealed a minor contribution, mainly localized in neuronal somas which co-store CGRP (Edvinsson L. et al., 2020). PACAP-38 is expressed in the TG of rat and man, and shown to co-localized with CGRP specifically in the neurons in TG (Frederiksen et al., 2018). In the walls of cerebral arteries there was a rich distribution of CGRP, but some perivascular fibers also contain substance P and PACAP-38 (Edvinsson and Uddman, 2005; Frederiksen et al., 2019). The dura mater with the middle meningeal artery (MMA) and its branches showed CGRP positive fibers but few containing substance P and essentially none with PACAP-38 (Edvinsson J. C. A. et al., 2020). In studies of the C-fiber distribution within the trigeminal nerve, there were C-fiber boutons being enriched with CGRP (Edvinsson et al., 2019); these also expressed some vesicular substance P but no PACAP-38 (Edvinsson J. C. A. et al., 2020). Based on these findings, co-release experiments were performed in different parts of the TGV system.

When stimulating the tissue preparations, it was observed that 60 mM K^+^ (depolarization) resulted in minor release of substance P from the dura mater but no release from the soma-poor or soma-rich samples (Edvinsson et al., 2021). However, there was abundant release from all three preparations of CGRP as measured in parallel. When adding the TRPV1 agonist capsaicin there was no observed release of substance P from the dura mater samples or soma-rich parts of the TG but a low release was detected from the soma-poor TG. In contrast, all three samples showed high and consistent release of CGRP following capsaicin treatment (Edvinsson et al., 2021). This pattern of release agrees well with the immunohistochemical expression of substance P and CGRP.

The PACAP-38 release data demonstrated that CGRP was released from all three parts of the TGV system; however, PACAP-38 was only released from the soma-rich part of the TG (with the neurons) and showed no differences if it was stimulated with 60 mM K^+^ or capsaicin (Edvinsson J. C. A. et al., 2020). This finding agrees well with the immunohistochemistry revealing that PACAP is only expressed in a subpopulation of CGRP positive neurons in the TG (Eftekhari et al., 2013; Frederiksen et al., 2018; Edvinsson J. C. A. et al., 2020). Taken together the data strongly suggest that the C-fiber boutons may function as local release sites.

Immunohistochemistry of the major blood vessels, cerebral and MMA, revealed some structural differences. Previous studies revealed a rich distribution of CGRP and substance P in cerebral vessel walls while the perivascular network of these fibers was comparatively sparse in MMA and in dura mater (Edvinsson et al., 1981, 1987). Only few PACAP-38 immunoreactive fibers were observed. All these fibers were observed in the adventitia, sometimes near the outermost part of the medial layer with vascular smooth muscle (VSM) cells. This would represent the far end of the TGV system.

## Receptors for Calcitonin Gene-Related Peptide, Substance P, and Pituitary Adenylyl Cyclase-Activating Polypeptide in the Trigeminovascular System and in Intracranial Vessel Walls

Numerous experiments on various types of vascular preparations, *in vivo* as well as *in vitro*, have been performed (Edvinsson and Uddman, 2005). Since migraine is a human disorder this section will focus mainly on human material when possible. CGRP, amylin, and adrenomedullin relax human MMA/branches with CGRP being strongest and the most potent of these agonists. Human cerebral arteries respond even more potently to CGRP than the MMA (Sams et al., 2000; Edvinsson et al., 2007). In perfused middle cerebral artery (MCA) from rat neither CGRP, amylin nor adrenomedullin pass cerebral artery endothelium when given luminally due to the presence of the BBB. However, when given abluminal these peptides elicited concentration-dependent dilatation that could be blocked by gepants or mAbs (Edvinsson et al., 2007).

The parasympathetic peptides: vasoactive intestinal peptide (VIP), peptide histidine isoleucine (PHI), peptide histidine methionine (PHM), and PACAP-38, are members of the secretin/glucagon superfamily of peptides, and have been shown to relax in a concentration-dependent manner, human temporal, meningeal, and cerebral arteries (Jansen et al., 1992; Grande et al., 2014). In a study on the MMA (Chan et al., 2011) it was reported that VIP (pEC_50_ = 7.4) was more potent than PACAP (<6.9); in addition, neither VPAC_1_ nor PAC_1_ receptor blockers antagonized the vasodilator responses to PACAP-38 in the MMA. This is probably due to low blocking capacity in the concentrations used, because both cerebral and MMAs contain mRNA for all three receptors (Knutsson and Edvinsson, 2002; Chan et al., 2011). In this work 30 mM K^+^ was used for pre-contraction and substance P to evaluate vasodilator capacity and endothelial function (Chan et al., 2011). Substance P is a strong vasodilator that acts *via* endothelial receptors to induce formation of nitric oxide (NO) which in turn activates guanylyl cyclase in the vascular smooth muscle cells (VSMCs). While several studies with the “infusion model” used NO or an NO donor (Lassen et al., 2003) to elicit migraine-like headache, there is no study on the effects of substance P in this paradigm (Ashina et al., 2017).

The pharmacological approach, has shown that VIP is a more potent vasodilator compared to PACAP-38 in man, both *in vivo* (Rahmann et al., 2008; Schytz et al., 2009) and *in vitro* on human MMA (Chan et al., 2011). Subsequent work on the dichotomy of VIP/PACAP infusions with magnetic resonance angiography showed dilatation of the extracranial superficial temporal artery but no effect on the diameter of the intracranial arteries, MMA, or cerebral arteries (Amin et al., 2014). Based on these findings, Tfelt-Hansen suggested that PACAP-38 might have an effect on sites within the CNS. We would as an alternative propose an effect on the sensory afferent nerves since there are receptors for both peptides available (Knutsson and Edvinsson, 2002; Chan et al., 2011). More precisely, the PAC_1_ receptor is present in the TG satellite cells but not on the Aδ-fibers which explains why the clinical studies with a specific mAb toward the PAC_1_ receptor was negative in prevention of migraine (Ashina et al., 2021a). The role of PACAP-38 in the TGV is still under scrutiny and the recently developed mAbs toward PACAP-38 will give more final answers on the involvement of PACAP in migraine pathophysiology (Moldovan Loomis et al., 2019).

## The Nodes of Ranvier – A Place for Interaction Between C- and Aδ-Fibers

**FIGURE 2:**
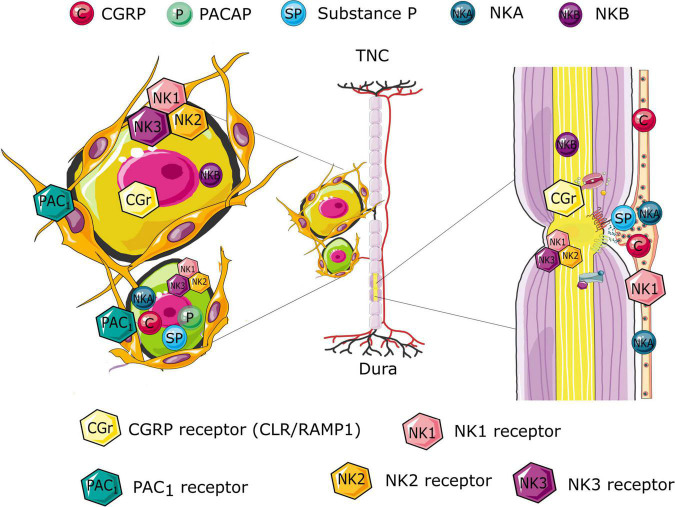
Schematic illustration of the differential distribution of the CGRP, pituitary adenylyl cyclase-activating polypeptide (PACAP), and Neurokinin signaling compounds in the trigeminal system. The illustration suggests possible site of origin if the various peptides and their associated release. Further, the receptor expression highlights possible receptor targets, following the peptide release. The expression is based on immunology findings from the following studies (Edvinsson et al., 2019, 2021; Edvinsson J. C. A. et al., 2020; Edvinsson L. et al., 2020).

Nodes of Ranvier are essential for the regeneration of diminishing action potentials in the nervous system (saltatory conduction). Although they have traditionally been regarded as passive contribution to action potential propagation, more recent work has suggest that they may have an active role in regulating neuronal excitability (Edvinsson et al., 2019). The nodal propagation has been suggested to be plastic and hence could excitingly be explaining the sensitization process which is a key element in migraine symptomatology. With the use of different antibodies toward elements of the nodal region we have shown with clarity their dense expression in the trigeminal nerve (Figure 2), both within the ganglion and in the nerve fibers going in central and peripheral directions (Edvinsson et al., 2019).

Neurons of the TG project with either thin, unmyelinated C-fibers or larger, myelinated A-fibers. While C-fibers are classically categorized, based on conduction velocity, as a single type, the A-fibers are of three subtypes: Aα, Aβ, and Aδ. Aα-fibers have the largest diameter (13–20 μm) with a conduction velocity of 80–120 m/s, followed by Aβ-fibers (6–12 μm; 30–70 m/s) and last the Aδ-fibers (1–5 μm; 3–30 m/s), while the C-fibers are slow conductors (0.2–1.5 μm; 0.5–2 m/s). This categorization may vary slightly between different species which makes it difficult to morphologically determine or differentiate a small Aβ or a larger Aδ-fiber. C-fibers and Aδ-fibers make up most axons within the trigeminal system and are related to nociceptive signaling. C-fibers are responsible for slow, dull burning pain while Aδ-fibers signal a quicker, sharper pain. This makes them important biological structures to consider when studying headache, pain, or allodynia related to the trigeminal system. Electrophysiology at different parts of the TGV system, mainly the neurons, shows two types of signaling velocities, one is the C-fibers and the other often categorized as Aδ-fiber neurons.

Stimulation of these nerve fibers at TG regions lacking neuronal somas results in the release of CGRP, which supports release from the C-fibers. In relation to the nodes of Ranvier in the trigeminal system we have shown that the C-fiber boutons contain large amounts of CGRP (Edvinsson et al., 2019) and smaller amounts of substance P (Edvinsson et al., 2021) but no PACAP (Edvinsson J. C. A. et al., 2020). This would favor the view that the first two neuropeptides may have a role albeit perhaps at different degrees.

The release from extra synaptic regions such as the varicosities, also referred to as “en-passant boutons,” are characteristic of presynaptic release sites in peripheral nerves (Smolen, 1988). We have postulated that CGRP and substance P could be released from these varicosities, although these areas are not classically understood to be synaptic. There are increasing evidence that neuropeptide release does not typically occur at defined synapses (Torrealba and Carrasco, 2004). Neuropeptides, such as CGRP, are typically stored in large dense-core vesicles, which are typically stored away from the presynaptic membrane, which for example has been shown for orexin (Torrealba et al., 2003). Other exocytosis sites have been suggested also due to the location of receptors for signaling molecules outside of typical synaptic regions (Trueta and De-Miguel, 2012). Although many of the mechanisms are common mechanisms, there are other aspects that are more similar to those of exocytosis from excitable endocrine cells. One such example is related to substance P, where exocytosis from the somata of dorsal root ganglion neurons has been demonstrated (Huang and Neher, 1996). This highlights the possible extra synaptic release of neuropeptides, and we suggest that the varicosities at the nodes of Ranvier provides such a possible release site (Edvinsson et al., 2019).

On the adjacent nodes of Ranvier where the myelin sheath is absent, and the Aδ-fiber is exposed we have demonstrated the presence of the CGRP receptor (Edvinsson et al., 2019) and recently also the neurokinin 1 receptor (NK1R; Edvinsson et al., 2021). This opens for diffusion of the two peptides to reach targets on the Aδ-fiber. The presence of adenylyl cyclase and protein kinase A further add to the activity of this pathway in the Aδ-fiber presumably modifying the activity of potassium K^+^ and sodium Na^+^ channels and the saltatory activity within the Aδ-fiber and the pain signaling. The Nav channels have received recent attention in light of their strong link to pain transmission (Hameed, 2019). In the nodes of Ranvier both Nav1.6, Nav1.7, and Nav1.8 have been shown to be expressed specifically in the nodal structure (Caldwell et al., 2000; Devaux and Scherer, 2005; Black et al., 2012), although Nav1.8 typically only do so in disease model (Devaux and Scherer, 2005). For Nav1.6 the dependency of PKA is present but does not have a major influence (Chen et al., 2008). However, Nav1.7 and Nav1.8, when expressed in Xenopus oocytes, are differentially regulated by PKA and PKC. Focusing on the cAMP pathway, PKA activation resulted in a dose-dependent potentiation of Nav1.8 currents and an attenuation of Nav1.7 currents (Vijayaragavan et al., 2004). This work is in its infancy but could potentially add novel understanding to processes of sensitization and as a drug target for headache disorders.

## Human Data in Light of the Node of Ranvier

Although the infusion model (Ashina et al., 2017) as a basis of triggering migraine-pain appears to be based primarily on the vascular theory of migraine more specifically on vasodilatation of cranial arteries, we suggest that some of the data can be extrapolated to the nodes of Ranvier. We have previously proposed that some of these data, e.g., when applying a K^+^ channel opener, could lead to long lasting hyperpolarization that activates Hyperpolarization-activated Cyclic Nucleotide-gated (HCN) Channels (Haanes and Edvinsson, 2020). The fact that both cAMP and cGMP can activate these channels (Momin et al., 2008) and lead to increases in the open-probability and augmented neuronal excitability and firing of the neurons (Momin et al., 2008) supports this notion. Furthermore, injection of CGRP or other migraine triggering molecules, also share the common cAMP pathway (Ashina et al., 2021b). We hypothesize that the key regulators of the node of Ranvier are the cyclic nucleotides, and indeed we have localized the downstream PKA to the nodes (Edvinsson et al., 2019). It cannot be ruled out that local potassium or other secondary messengers following dilation also activate the C-fibers (Al-Karagholi et al., 2021) but we propose nodal or neuronal activation could be the key to understanding the infusion model. It is further worth pointing out that only passive vasodilation of cranial arteries is not the likely inducer of CGRP release, as it was shown that the TGV reflex demonstrated that vasoconstriction of cortex pial arterioles results in activation of the TGV system and release of the stored vasoactive peptides (McCulloch et al., 1986; Edvinsson et al., 1990).

## The Remak Bundles on C-Fibers

Schwann cells can either be “myelinating” or “non-myelinating” meaning that they are either specialized in insulating a portion of an A-fiber with myelin, or mainly providing structural stability for a bundle of C-fibers (called Remak bundles) (Aguayo et al., 1976). The myelin sheath provides isolation making the transmission of an electrical signal move faster through the myelinated segments of the axon. Without the nodes of Ranvier, a signal traveling too far would lose energy and fade out or be diminished before reaching its intended terminal, making the signal unable to proliferate. This process is an important prerequisite for adequate function of nerves both in the CNS and in the PNS. As an alternative to the myelin coverings of the Aδ-fibers in the TGV system the C-fibers have been suggested to be partly covered by so-called non-myelinating Schwann cells. The main population of axons surrounded by non-myelinating Schwann cells are the small nociceptive C-type of axons. This also applies for postganglionic sympathetic axons, and some of the preganglionic sympathetic and parasympathetic fibers (Griffin and Thompson, 2008). Remak non-myelinating Schwann cells envelop sections of the axons within troughs on their surface. To our knowledge, no specific antibody exists for differentiating between myelinating and non-myelinating Schwann cells. During our immunohistochemical studies we have therefore, so far, not been able to identify these structures. If Remak bundles are abundant in the TGV system it could limit some of the C-fibers from signaling to adjacent fibers (Edvinsson et al., 2019). However, in our experimental studies on soma-poor trigeminal nerves we observed strong release of CGRP which indicates that peptides located in C-fibers can diffuse outside the Remak bundles and act on the CGRP receptors located on the Aδ-fibers at the nodes of Ranvier. The released CGRP can also be measured in cranial vein plasma as shown in several species including man. With existing information from literature, the Remak bundles usually refer to groups of C-fibers and not, as we often see them, as single C-fibers in the TGV system (Faroni et al., 2014; Miyamoto et al., 2017).

Calcitonin gene-related peptide has been shown to lead to increases in iNOS expression (Vause and Durham, 2009). And although not in the scope of the current review, there is also extensive research showing that nitric oxide can modulate action potentials. Relating to the node of Ranvier, iNOS immunoreactivity has been shown to be expressed in Schwann cells of peripheral nerves and was particularly enriched at the paranodal regions of the nodes of Ranvier (Chen et al., 2010). In addition, activation of cGMP might link to membrane channel activities such as the HCN channel (Momin et al., 2008), which deserves further research.

## The Nerve Root Entry Zone – The Redlich-Obersteiner Zone

Ever since its discovery 150 years ago much attention has been directed to the Redlich-Obersteiner zone (Savvaidou and Triarhou, 2016). This region exists for all cranial nerves and hence is postulated to be involved in many cranial nerve disorders such as trigeminal neuralgia (Nomura et al., 2019) and other clinical applications (Xenellis and Linthicum, 2003). It represents the” junction zone” of glia and Schwann sheath of the cranial nerves. In this zone, the Schwann cells are abruptly replaced by the more numerous oligodendrocytes and a clearly visible zone between the PNS and the CNS appear. This area is believed to be linked to trigeminal neuralgia caused by compression of the nerve root (Jannetta, 1967). However, many of the functional details remains to be understood; how the PNS and CNS communicates, how the functionality of the BBB is upheld in this region and what role the change of glial cells may have on axonal signaling. Possible cross-talk among fibers in this region may be crucial in understanding parts of the migraine pathology.

## Conclusion

Here we have discussed the possibility that the nodes of Ranvier, expressed in most parts of the CNS and the PNS may be involved as an important mechanism to modify signaling in the trigeminal system and perhaps even have a role in the sensitization process. CGRP and its receptor represent the most expressed system while substance P is less densely supplied but still may be involved in the headache processes. PACAP has been suggested as a putative novel molecule in headache disorders but future work remains to unravel its role in migraine. While there was no PACAP in the trigeminal nerves we observed some expression in trigeminal ganglion cells and the PAC_1_ receptor in satellite glial cells in the ganglion. There remain numerous questions to address also for the other members of the CGRP family of peptides and their receptors (Edvinsson L. et al., 2020).

## Author Contributions

All authors listed have made a substantial, direct, and intellectual contribution to the work, and approved it for publication.

## Conflict of Interest

The authors declare that the research was conducted in the absence of any commercial or financial relationships that could be construed as a potential conflict of interest.

## Publisher’s Note

All claims expressed in this article are solely those of the authors and do not necessarily represent those of their affiliated organizations, or those of the publisher, the editors and the reviewers. Any product that may be evaluated in this article, or claim that may be made by its manufacturer, is not guaranteed or endorsed by the publisher.
